# Beliefs about Vaccinations: Comparing a Sample from a Medical School to That from the General Population

**DOI:** 10.3390/ijerph15040620

**Published:** 2018-03-28

**Authors:** Lauren E. Latella, Robert J. McAuley, Mitchell Rabinowitz

**Affiliations:** 1 Graduate School of Education, Fordham University, New York, NY 10023, USA; mrabinowitz@fordham.edu; 2 Institutional Effectiveness and Technology, Oakland University William Beaumont School of Medicine, Rochester, MI 48309-4482, USA; mcauley@oakland.edu

**Keywords:** expert knowledge, illusion of uniqueness, communication, vaccinations, consensus bias, medical affiliates

## Abstract

The current study compares health care professionals’ beliefs about vaccination statements with the beliefs of a sample of individuals from the general population. Students and faculty within a medical school (*n* = 58) and a sample from the general population in the United States (*n* = 177) were surveyed regarding their beliefs about vaccinations. Participants evaluated statements about vaccinations (both supporting and opposing), and indicated whether they thought the general population would agree with them. Overall, it was found that subjects in both populations agreed with statements supporting vaccination over opposing statements, but the general population was more likely to categorize the supporting statements as beliefs rather than facts. Additionally, there was little consensus within each population as to which statements were considered facts versus beliefs. Both groups underestimated the number of people that would agree with them; however, the medical affiliates showed the effect significantly more. Implications for medical education and health communication are discussed.

## 1. Introduction

Recently, a number of parents have taken alternative approaches to vaccinating their children by either delaying vaccinations or skipping them all together [1,2,3]. Consequently, several diseases that were nearly eradicated such as whooping cough, measles, and diphtheria have re-emerged [4,5]. Research has shown that parents are increasingly sensitive to the perceived harms and benefits of vaccinating their children which results in differences in their overall perceptions regarding the benefits of vaccinations [6,7]. The re-emergence of vaccine-preventable diseases has sparked public health interest in exploring people’s beliefs about the positive and negative outcomes of childhood vaccinations [8]. 

Literature has highlighted that parental health-related decision making is influenced by the effectiveness of paediatricians’ communication about childhood vaccinations [2,3,9,10,11,12,13]. More specifically, Opel and colleagues [9] have illustrated that physician communication regarding vaccinations is associated with parents abiding by the prescribed childhood vaccination schedule. Therefore, research has focused on evaluating provider–parent vaccination discussions to attempt to pinpoint effective communication styles and strategies [9,14]. Additionally, a systematic review on parental attitudes and beliefs regarding childhood vaccinations pinpointed poor communication skills and physician distrust as prime barriers in timely vaccinations [15].

Communication research has been a salient field in educational and research institutions [16,17,18]. Effective communication is dependent on understanding the intended audience. There are specific psycholinguistic elements that a communicator must consider to ensure that the audience will be able to appropriately comprehend the information provided [19,20,21]. Grice, [19] for example, postulates that an effective and efficient communicator should only provide information that is succinct, necessary, non-ambiguous, and evidence based. Further, Clark and Haviland [21] state that communicators should continuously monitor audience understanding and construct their semantic communication accordingly. In the current study, we explicitly compared a group of health care affiliates’ (i.e., students and faculty from one medical school) beliefs about the positives and negatives of vaccinations with those of a sample group obtained from the general population. We also sought to explore the two groups’ perceptions of what others thought of the information.

Research has shown that experts often fail to consider their audience’s scope of knowledge because it is difficult for experts to anticipate the audience members’ differing degrees of baseline knowledge [22,23,24]. However, when experts model their knowledge to better match their audience’s baseline knowledge, the experts can communicate more efficiently and effectively. When experts communicate effectively, the audience understands and retains the message better [22,25]. Experts can accomplish this by focusing the semantic content of their discussions on essential concepts rather than unnecessary technical information [22,25]. Laypersons take away more information when the meaning of the concepts is made salient or when experts are more aware of what people believe or do not believe.

However, appropriately recognizing and adjusting semantic content to reflect a lay audience’s understanding is a challenging task. Experts often exhibit biases when communicating with lay persons [26]. Nickerson and colleagues [26] describe three hypotheses for evaluating individuals’ communication styles—correspondence, overestimation, and expertise. The correspondence hypothesis states that individuals display a false consensus effect when estimating their level of knowledge as compared to others. A false consensus effect happens when people overestimate that people believe or know something. Therefore, if an individual has knowledge about a given subject, he or she is more likely to attribute this knowledge to others. Regarding the overestimation hypothesis, Nickerson et al. [26] suggest that people, in general, overestimate the commonality of their knowledge; therefore, they believe that others have the same level of knowledge as themselves.

Support for Nickerson et al.’s [26] hypotheses have been mixed in the literature. Fussell and Krauss [27] confirmed Nickerson and colleagues’ overestimation and correspondence hypotheses. They found that experts substantially overestimated their knowledge and abilities regardless of whether the experts knew the subject matter. The researchers also reported that laypersons were more likely to estimate other’s knowledge in correspondence to their own [27,28]. Similarly, Nickerson et al. [26] reported that individuals who possessed knowledge in a given domain were more likely to overestimate the commonality of their knowledge. In an attempt to provide further support for these hypotheses, Bromme, Rambow, and Nückles [29] created several studies to examine the differences in estimation of knowledge between laypersons and experts. However, their findings did not provide support for the expertise hypothesis. The experts in these studies were significantly more cautious in their estimations compared to the laypersons’ estimations. They tended to underestimate the knowledge of the laypersons and viewed their specialist knowledge as unique. Bromme et al.’s [29] findings did, however, confirm the overestimation hypothesis. 

The overestimation of the layperson’s knowledge can be problematic in the delivery of crucial information. Specifically, Wittwer, Nückles, and Renkl [30] found that laypersons’ learning was impaired when experts overestimated the audience’s baseline level of knowledge of the topic. Individuals had greater difficulty comprehending the information. This generated more questions for clarity. Often, an expert’s responses were not constructed to ensure that the laypersons understood the message, and resulted in inefficient and ineffective communication [30]. Therefore, it is important to evaluate potential discrepancies between physicians’ and patients’ beliefs. If physicians are inaccurate when estimating the baseline knowledge of their patients, it is possible that patients’ understanding of illness, adherence to treatment, and medical decision making will be impaired.

This mismatch may lead to increased resistance to the use of preventive health measures, such as childhood vaccinations, as overestimations may influence a discrepancy between physician-patient shared understanding about the benefits and risks of childhood vaccinations. When there is a mismatch in shared beliefs between physicians and parents in relation to vaccinations, research has shown that parents tend rely less on scientific information regarding vaccinations [5,6]. Additionally, effective communication fosters trust in physicians’ recommendations. A trusting physician-patient relationship has played a pivotal role in the decision-making of new mothers regarding the willingness and timeliness of vaccinating their children [31,32].

Specific communication skills such as acknowledging parents’ concerns, using non-jargon language, and checking for parental understanding have been highlighted as beneficial strategies during conversations regarding the risks and benefits of childhood vaccinations [33]. In addition, Connors and colleagues [32] conducted a systematic review on physician–parent communication regarding childhood vaccinations. They found that vaccine-hesitant parents were more receptive to individualized discussions with their children’s physicians which fostered an open format in which the parents felt understood and validated. However, it is important to note that parents were more likely to agree to child immunizations in a timely fashion when their physicians initiated the vaccination conversation in a presumptive manner [9,14]. Evaluating the discrepancy between medical affiliates’ and the general population’s beliefs about vaccinations may lead to pinpointing the accuracy of the population’s perceptions of consensual beliefs. Research has identified that individuals who overestimate the extent to which the general population share their beliefs exhibit the ‘truly false consensus effect’; whereas, individuals who underestimate the extent to which the general population may hold different views, exhibit ‘an illusion of uniqueness’ [34,35,36]. Further identifying how healthcare providers and medical consumers (i.e., general population) perceive the benefits and risks of childhood vaccinations can provide opportunities to explore how medical affiliates are delivering messages about domain-specific knowledge to a lay audience. 

The current study attempts to address five research questions:

1. To what extent do people agree with supporting (pro) and opposing (anti) statements regarding vaccinations?
2. Do people categorize these statements as facts or beliefs? Rabinowitz and colleagues [8] argued that categorizing a statement as a fact, as opposed to a belief, is related to people’s views of social norms; people tend to categorize statements as facts when they think that most people would agree with it. Therefore, the categorization question goes beyond just whether people agree with a statement or not.
3. Do people agree as to what is a fact and what is a belief?
4. Are people accurate in predicting what the general adult population would believe?
5. Do the answers to the above four questions vary between medical affiliates (i.e., a population more versed in domain-specific knowledge) and the general population?

Consistent with previous findings which evaluated the general population’s beliefs regarding childhood vaccinations, it is hypothesized that the majority of participants will be more likely to agree with the pro-vaccination statements and categorize them as facts rather than as beliefs [8]. As widely illustrated in the medical field’s firm recommendations of childhood vaccinations [1,2,3,4,5,6,7], it is anticipated that medical affiliates will report higher levels of agreement and categorize the statements as facts more than the general population. Congruent with previous findings [8], it is expected that overall, there will be little agreement as to which statements should be considered facts versus beliefs. However, it is anticipated that consensual beliefs will be stronger among the medical population due to their expertise knowledge in the subject matter, as depicted in the literature on expertise knowledge [17,18,19,20,21,22]. Lastly, based on Nickerson and colleagues’ theory [26], it is hypothesized that medical affiliates will overestimate the general population’s beliefs about vaccinations, whereas the general population will be more conservative in their estimations.

## 2. Method

### Materials and Procedure

For the general population group, the stimulus materials were presented by a link to Survey Monkey through Mechanical Turk. For the medical affiliates, the stimulus materials were obtained through an emailed Survey Monkey link. The general population participants received 25 cents in their Amazon account as compensation for completing the survey. The medical affiliates did not receive any form of compensation. On the first page of the survey, participants were presented with a consent form which outlined the purpose of the study and brief instructions regarding the study tasks. Once consent was obtained, the participants were provided with a demographic survey to collect information about their gender, age, education/medical level, ethnicity, primary language, and state of residency. 

To familiarize participants with the fact/belief categorization task and to also collect data as a comparison check for the vaccination content, a list of 20 statements about general world knowledge was presented. These statements were used in prior research [8,37], and consisted of 10 statements that were generally accepted as facts (e.g., “A hammer is a tool used to pound nails”), and 10 statements that were generally accepted as beliefs (e.g., “sleeping with the windows open is good for you”). See Appendix A for a list of the 20 general statements. The statements were presented to the participants in a random order. For each statement, participants were asked to answer the following questions: (1) “How strongly do you agree with this statement?” (4-point Likert scale: *1 = strongly disagree*, *2 = disagree*, *3 = agree*, *4 = strongly agree*); (2) “What percentage of the general adult population would agree with this statement?” (5-point scale: *0%*, *25%*, *50%*, *75%*, or *100%*); and (3) “Is this statement a fact or belief?” (dichotomous response). 

Following these statements, the participants completed a similar task with respect to 20 statements pertaining to childhood vaccination, including 10 pro-vaccination and 10 anti-vaccination statements, presented in random order. An example of a pro-vaccination statement is “Vaccinations are necessary for eliminating vaccine-preventable diseases”, and an example of an anti-vaccination statement is “Vaccinations can have serious side effects that cause more harm than some of the diseases that they are supposed to prevent”. See Appendix B for the vaccination statements. The vaccination statements were derived from websites and media sources, including vaccines.procon.org, which featured vaccination related information from the Center for Disease Control, as well as from parents.com, kidshealth.org, huffingtonpost.com, NYTimes.com, Harvard Health Publications, the U.S. Department of Health and Human Services website, thehealthyhomeeconomist.com, and DrFeder.com (a homeopathic Pediatrician’s website). For each of the 20 vaccination statements, participants were asked the same three questions as presented in the general statement section. Participants responded at their own pace. The entire survey took an average of 11 minutes to complete. Recruitment methods and study procedures were approved by the Fordham University IRB, #IRB-14-12-MR-101 and Oakland University Medical School IRB, IRB #716034-1.

## 3. Results

The relevant data for the general statement analyses can be found in the online supplemental information index. Also, an initial analysis of all dependent variables was conducted comparing medical students with the medical faculty. No significant differences were observed for any of the dependent variables to these two groups were merged into a group of medical affiliates.

### 3.1. Participants

The participants in this study were comprised of 235 individuals over the age of 18. Of the 235 participants, 58 individuals (15 students and 43 faculty) were medical affiliates from the Oakland University William Beaumont School of Medicine. The remaining 177 participants were obtained through the general population using Mechanical Turk. Five additional participants were originally recruited through Mechanical Turk; however, they were excluded from statistical analysis due to suspicious or abnormal response patterns. Four out of the five participants agreed with every statement, and the remaining one participant responded in a disagreement rate that was more than three standard deviations from the sample mean. Four additional medical school affiliates were eliminated from the final sample as they did not complete the task fully. The mean age for medical affiliates was 30.4 years with a range of 21–66 years old. Similarly, the mean age for the general population was 33.6 years with a range of 18–72 years old. All participants were primarily English-speaking. Medical affiliates consisted entirely of individuals who resided in Michigan at the time of data collection whereas the general population comprised of individuals from 40 different states. See Table 1 for participant characteristics. 

### 3.2. Agreement for the Vaccination Statements

Each participants’ overall agreement was calculated for the vaccination statements. A value of 1 was given for each statement the participants claimed that they agreed with, and a value of 0 reflected no agreement with the statement. The average agreement was calculated by total agreement across the vaccination statements divided by 20 statements. A repeated measures mixed design (between subjects–population; within subjects–statement type) ANOVA was conducted to evaluate whether there was a significant difference in regard to level of agreement between the two populations and two types of statements. There was a significant effect of statement type *F*(1, 233) = 339.64, *p* < 0.001, *η_p_*^2^ = 0.593. Both groups were more likely to agree with the pro-vaccination statements (*as* compared to the anti-vaccination statements (*M*_medical affiliates_ = 0.31, *SD* = 0.17; *M*_general population_ = 0.42, *SD* = 0.29). There was a nonsignificant effect of population type *F*(1, 233) = 3.07, *p* = 0.081; medical affiliates = 62%, general population = 65%. There was also a significant interaction between statement and population type, *F*(1, 233) = 9.18, *p* = 0.003, *η_p_*^2^ = 0.038. Even though both groups indicated that the anti-vaccination statements were less agreeable, the medical affiliates were significantly less likely to agree with the anti-vaccination statements. 

### 3.3. Categorization of Facts and Beliefs

Each participants’ fact categorization was calculated for the 20 vaccination statements. If participants selected that the statement was a fact, the statement received a score of a 1; whereas if the participants indicated that the statement was a belief, the statement received a score of a 0. The average of all the responses was determined to determine an average fact versus belief score for each statement. See Table 2 for the average fact versus belief scores for each vaccination statement for the general population and the medical affiliates. 

A repeated measures mixed design ANOVA was conducted to assess the differences between the general population’s categorization of vaccination statements and the medical affiliates’ categorization of vaccination statements. There was a significant effect of statement type *F*(1, 233) = 16.66, *p* < 0.001, *η_p_*^2^ = 0.579. Both groups were more likely to categorize pro-vaccination statements as facts (*M*_medical affiliates_ = 0.79, *SD* = 0.22; *M*_general population_ = 0.60, *SD* = 0.32 and anti-vaccination statements as beliefs (*M*_medical affiliates_ = 0.26, *SD* = 0.16; *M*_general population_ = 0.26, *SD* = 0.21). There was also a significant effect of population type *F*(1, 233) = 10.66, *p* = 0.001; medical affiliates = 52.35%, general population = 42.65%. There was a significant interaction between statement and population type, *F*(1, 233) = 15.47, *p* < 0.001, *η_p_*^2^ = 0.062. Even though both groups categorized the pro-vaccination statements as facts, the medical affiliates were significantly more likely to categorize more pro-vaccination statements as facts. 

### 3.4. Do People Agree as to What Is a Fact and What Is a Belief?

To determine if the medical and the general population had consensus, or agreed with each other as to which vaccination statements were facts and beliefs, fact categorization scores were computed. Each statement by a participant received a score of 1 if they categorized the statement as a fact, and a score of 0 if they categorized the statement as a belief, and the average score for each statement was computed. Therefore, if all the participants indicated that the statements were “facts”, the average fact categorization for the statement would be 1. An average score of 0 would infer perfect agreement that a statement is a belief. A score of 0.5 would indicate that 50% of participants categorized the statement as a belief and 50% categorized the statement as a fact; therefore, representing no consensus. 

The average consensus values for each of the vaccination statements were converted into scores that ranged from 0 to 0.4. These scores were obtained by assigning a value based on how far away a statement was from agreement (0 or 1). The number of statements that had average values ranging from 0–0.09 or from 0.9–1 were multiplied by 0. The number of statements that had average values from 0.1–0.19 and 0.8–0.89 were multiplied by 0.1. The number of statements that had average values ranging from 0.2–0.29 or 0.7–0.79 were multiplied by 0.2. The number of statements that had average values ranging from 0.3–0.39 and 0.6–0.69 were multiplied by 0.3. Lastly, the number of statements that had average values ranging from 0.4–0.49 and 0.5–0.59 were multiplied by 0.4. If perfect consensus among all participants across all statements was achieved, the average consensus value would equal 0. 

**Figure 1:**
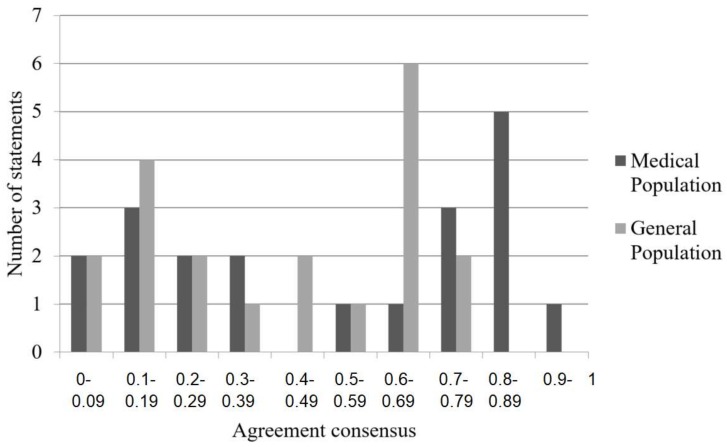
Number of statements as a function of average consensus for the vaccination statements.

The consensus values were then compared to the expected value of 0.2 (i.e., the value associated with random selection). Consensus values that were significantly less than 0.2 were considered as representing consensus among the participants as to which statements were categorized as facts and which statements were characterized as beliefs [23]. See Figure 1 for the number of vaccination statements plotted as a function of the average consensus values. It is interesting to compare this graph, with that generated through analysis of the general materials where there is considerable consensus. That graph can be found in the supplemental materials.

Independent t-tests were conducted to determine if there was consensus among the two groups. The medical affiliates’ consensus averages were not significantly different from 0.2 (*M* = 0.12, *SD* = 0.11), *t*(57) = −1.83, *p* = 0.083; therefore, there was no consensus between which statements were categorized as facts and which statements were categorized as beliefs within the medical affiliates. Similarly, there was no consensus within the general population as to which vaccination statements were categorized as facts versus beliefs (*M* = 0.23, *SD* = 0.13), *t*(177) = 0.89, *p* = 0.383. 

### 3.5. Accuracy of Predictions

Participants were asked to predict the percentage of people that would agree with each statement. The actual and predicted values of agreement among the vaccinations statements were calculated. Accuracy values were calculated by taking the difference between each participants’ agreement estimates for the general population and the average of the agreement calculated from all the participants for each statement. A correlation with Fisher correction was then calculated between the participants level of agreement and the accuracy of their prediction. This measure would show whether people were overestimating the level of agreement of their beliefs with that of others (the truly false consensus effect) or underestimated the level of agreement (the illusion of uniqueness).

Independent t-tests were computed for both the medical affiliates and the general population to observe if the accuracy values significantly differed from 0 (*M* = −0.539; *SD* = 0.38; *M* = −0.289, *SD* = 0.40, respectively). The accuracy values for the medical affiliates did significantly differ from 0, medical: *t*(57) = −10.68, *p* < 0.001, and for the general population: *t*(176) = −9.545, *p* < 0.001. Next, an independent t-test was computed to determine if there were significant differences in accuracy predictions between the two population groups. Both groups showed illusion of uniqueness; however, medical affiliates were significantly more likely to underestimate than the general population, *t*(228) = −4.35, *p* < 0.001. 

## 4. Discussion

The re-emergence of vaccine-preventable diseases has caused worry within the medical community about how to better tailor public-health interventions [5]. The continuous debates about whether the benefits of immunizing young children against specific diseases outweigh the potential risks have been a popular topic in the media. To gain further insight as to how people perceive the positives and negatives of vaccinations, the primary goal of this study was to determine whether people’s beliefs pertaining to childhood vaccination and their beliefs about what other people think about the evidence vary as a function of population type (i.e., medical affiliates and the general public). The results of our study suggest that the two populations hold relatively similar views about vaccinations; however, the medical population leans a bit more strongly toward agreeing with the supporting vaccination statements and categorizing these statements as factual information. 

Since the data suggests that both the general population and the medical affiliates agree with the pro-vaccination statements, medical affiliates do not necessarily have to emphasize the positives of childhood vaccinations. Contrastingly, the study illustrates that the general public are more likely to agree with the anti-vaccination statements compared to medical affiliates. This discrepancy suggests that clinicians should spend time to focus on countering parents’ anti-vaccination ideals during their clinical visits. This shift in communication style may address the variation between medical affiliates and the general population in terms of agreement with the anti-vaccination statements. Furthermore, it is likely that the general population’s perceptions of the negative effects of vaccinations may be lessened and anti-vaccination parents would be encouraged to accept the benefits of vaccinations when their clinicians focus their attention on the parents’ concerns. 

This is not necessarily a novel idea, as the Centers for Disease Control and Prevention (CDC) [38] have highlighted the importance of addressing parents’ negative perceptions of childhood vaccinations and both acknowledging and minimizing the risks of vaccinations. The CDC poses several questions that parents may bring up at clinical visits that clinicians should take into consideration. Potential questions include whether vaccines cause autism, and the risks, side effects, and unknown serious adverse events of certain vaccinations while considering the number, timeline, and vaccination ingredients [38]. 

Despite a breadth of research studies that have evaluated effective strategies for physician–parent dialogues regarding childhood immunizations, researchers note that the findings have only indirectly provided insight into communication skills [14,15,16,17,18]. It is possible that communication skills training programs for newly trained medical affiliates are neglecting to consider the fundamentals of psycholinguistic communication elements in discussions between experts and laypersons. By highlighting the findings in this study, (i.e., medical affiliates have the tendency to underestimate laypersons’ shared beliefs) physician communication training programs may benefit from addressing physicians’ delivery of information [14,15,16] and assumptions of parents’ level of vaccination understanding. Additionally, physicians may benefit from acknowledging the discrepancy between their perception of the general population’s beliefs compared to the general population’s actual beliefs, as this may encourage physicians to reframe conversations with parents regarding childhood vaccinations. 

Additionally, our findings do not support any of the three hypotheses for evaluating experts’ communication proposed by Nickerson and colleagues [26]; rather they highlight an opposite result. It is likely that the medical affiliates are aware that their medical knowledge, particularly in relation to vaccinations, is specialist knowledge that is not commonly shared among the general population due to differences in their education and training. Therefore, experts may perceive their knowledge to be exclusive based on their own abilities [39]. Stehr and Ericson [40] postulated that social recognition is necessary when specialist knowledge is emphasized in relation to laypersons’ understanding of the subject matter. Thus, the illusion of uniqueness results when experts, such as the individuals within the medical affiliates, distinguish themselves from the general population and estimate laypersons’ knowledge.

This finding encourages researchers to question the naivety of people’s belief systems, and the continuum of agreement in relation to shared beliefs among those with domain-specific knowledge and the general population. The extent to which people take ownership of their thoughts related to their knowledge may influence how individuals deliver messages about specific information. Implications for holding strong beliefs and viewing oneself as having expert knowledge should be further researched.

There are various limitations of this research study that should be addressed. Specifically, both population samples were not large enough to make broader claims regarding the United States’ population. More specifically, the study focused only on one medical school which could potential skew the results for the medical affiliates. This population also represented a broad spectrum of medical expertise from novice medical students to expert clinicians. It is possible that the environment and culture of the medical school may influence students and professors to hold specific views about their knowledge and their perceptions of social norms, as well as their understanding of the benefits and risks of vaccinations. A more diverse sample would be necessary to make greater generalizations about the medical community’s consensual views on the information pertaining to vaccinations. Additionally, the results may have been skewed due to the potential discrepancies between the interpretations of the word “fact”, and the inability to ensure that vaccination knowledge is standardized within the medical population. 

Future research should examine various other medical school populations to further generalize the view points of the medical community. Similarly, to gain a better understanding of people’s perceptions of health-related information, additional medical topics should be explored to determine if the illusion of uniqueness is specific to vaccination topics or if it exists with other health-related decisions. It is possible that the focus on differing views on vaccinations in the social media may contribute to individuals’ varied opinions regarding the factual and mythical vaccination information. Further research evaluating the long-term efficacy and safety of present childhood vaccinations by comparing the health outcomes of vaccinated and unvaccinated children would provide greater support for the development of individuals’ perceptions of vaccinations [41]. Additional studies should be conducted to further examine reasons why there is a lack of consensus among those with expertise knowledge in a given field. Lastly, future research should evaluate whether expert communication about other health-related topics violates Nickerson and colleagues’ [26] hypotheses. It is possible that the medical population is a unique group of “experts”, and thus, their continuous interactions with laypersons encourages them to be socially cognizant of their perceptions of the laypersons’ understanding of medical knowledge, as proposed by Stehr and Ericson [40] and Marks [39].

## 5. Conclusions

Our findings support that both the general population and medical affiliates share similar views on the benefits of childhood vaccinations; however, the general population was more likely to indicate that pro-vaccination statements are merely beliefs rather than factual information. The medical health providers also were more likely to disagree with the anti-vaccination statements compared to the general population. Therefore, it can be deduced that the variation in perceptions regarding childhood immunizations lies within parents’ anti-vaccination beliefs. Therefore, it is suggested that clinicians should focus less on convincing parents of the benefits of childhood vaccinations and emphasize communication that counters anti-vaccination beliefs. 

Our study also highlights the minimal consensus within each population as to which statements were considered facts versus beliefs. More specifically, both groups underestimated the number of people that would agree with them. Interestingly, the medical affiliates showed this effect significantly more. Therefore, clinicians should be aware of the illusion of uniqueness bias that they hold regarding vaccinations and potentially other health-related decisions because health professionals are identified as the most important source of information on vaccinations for the general population [12]. Despite various public health interventions, such as media coverage and vaccination advertisements, if physicians are not advocating for vaccination coverage, parents are less likely to choose to vaccinate their child [42,43]. Understanding that medical affiliates underestimate the amount of the population that agrees with them may encourage physicians to re-evaluate how they deliver messages about childhood vaccinations.

## Figures and Tables

**Table 1:** Participant characteristics (*N* = 235 participants).

Characteristic	Medical Population *n = 58*	%	General Population *n* = 177	%
Gender				
Male	28	48.30	55	31.10
Female	30	51.70	122	68.90
Ethnicity				
Caucasian	48	82.8	125	76.3
Asian/Pacific Islander	7	12.1	15	9.5
African American	1	1.7	13	7.3
Hispanic	1	1.7	9	5.1
Other	1	1.7	11	2.8
Medical Level				
Professor	15	25.9	-	-
Student	43	74.1	-	-
Education Level				
High School Diploma	0	0	62	35
BA/BS	0	0	77	43.5
Master’s degree	0	0	27	15.3
Other	58	100	11	6.2

**Table 2:** Fact versus belief averages for each vaccination statement by population type.

**Supporting Statements**	**Medical Population (*M*)**	**General Population (*M*)**
Eighty to 90% of a population needs to be vaccinated in order for an entire community to be fully protected against a disease.	0.78	0.45
Delaying or refusing vaccinations leaves children unprotected against many dangerous diseases.	0.83	0.70
Vaccinations against dangerous disease have saved more lives than drugs, such as antibiotics in the late 20th century.	0.69	0.62
The amount of ingredients used to create vaccinations is safe.	0.26	0.29
It is very rare to have an adverse reaction to a vaccine.	0.85	0.72
Mothers who are vaccinated protect their unborn children from viruses that can cause birth defects, such as mental disabilities, heart problems, and hearing and vision loss.	0.26	0.19
Vaccinations are necessary for eliminating vaccine-preventable diseases.	0.77	0.62
Vaccinations mobilize antibodies and proteins that are mimicked in the body’s natural immune defenses.	0.91	0.70
There is no direct link between vaccinations and autism spectrum disorder or other mental disabilities.	0.79	0.61
Vaccinations go through a long process in order to determine whether or not it is safe and effective for public use.	0.86	0.74
**Opposing Statements**	**Medical Population (*M*)**	**General Population (*M*)**
The increased number of vaccinations prior to a child’s second birthday is the reason why there has been an increase in autism spectrum disorder in children.	0.05	0.05
Vaccinating a child before his/her immune system is fully developed can cause harm to that child.	0.85	0.53
Vaccinations can have serious side effects that cause more harm than some of the diseases that they are supposed to prevent.	0.35	0.28
Vaccines introduce toxic chemicals that are not found in the natural immune defenses.	0.57	0.39
Even if people are vaccinated, there is still a risk of contracting the disease that the vaccination was intended to protect against.	0.83	0.63
Vaccinations do not lead to life-long immunity, whereas contracting a disease, such as chicken pox does result in life-long immunity due to the body’s natural defense mechanisms.	0.35	0.40
An increase in hygiene and improved living conditions are the reasons why the prevalence of diseases have declined, rather than an increase in vaccinations	0.10	0.15
The use of aluminum in vaccinations is a risk for Alzheimer’s disease, dementia, and seizures.	0.16	0.20
Due to political ties and economic incentives, pharmaceutical companies are not trustworthy.	0.16	0.19
Vaccinations expose children to mercury through the use of thimerosal which is a reason for the rise in autism spectrum disorder.	0.05	0.09

## Supplementary Materials

The following are available online at http://www.mdpi.com/1660-4601/15/4/620/s1, Figure S1: Number of statements as a function of average consensus for the general statements, Table S1: Fact versus belief averages for each general statement by population type.

## Appendix A

General Statements

1. A hammer is primarily used to pound nails.
2. Children are happy and carefree.
3. Dogs are animals.
4. A pen is for writing.
5. The telephone is the greatest invention of all time.
6. There are three colors in the American flag.
7. There are seven days in a week.
8. Christmas is a holiday primarily for children.
9. Sleeping with the window open is good for you.
10. Thermometers are used to record temperature.
11. A driver’s license is required for driving a car.
12. Books may be borrowed from the library.
13. Rich people are happy people.
14. Cats are friendly animals.
15. Rock music has a bad influence on young people.
16. It is okay to lie.
17. The longer you stay in school the smarter you will be.
18. The earth revolves around the sun.
19. Comic strips are funny.
20. The shape of a ball is round.

## Appendix B

Vaccination Statements

Pro-Vaccination Statements

1. Eighty to 90% of a population needs to be vaccinated in order for an entire community to be fully protected against a disease.
2. Delaying or refusing vaccinations leaves children unprotected against many dangerous diseases.
3. Vaccinations against dangerous diseases have saved more lives than drugs in the late 20th century, such as the development and use of antibiotics.
4. The amount of ingredients used to create vaccinations is safe.
5. It is very rare to have an adverse reaction to a vaccine.
6. Mothers who are vaccinated protect their unborn children from viruses that can cause birth defects, such as mental disabilities, heart problems, and hearing and vision loss.
7. Vaccinations are necessary for eliminating vaccine-preventable diseases.
8. Vaccinations mobilize antibodies and proteins that are mimicked in the body’s natural immune defenses.
9. The creation of vaccinations consists of a long process in order to determine whether or not it is safe and effective for public use.
10. There is no direct link between vaccinations and Autism Spectrum Disorder or other mental disabilities.

Anti-Vaccination Statements

1. The increased number of vaccinations prior to a child’s second birthday is the reason why there has been an increase in Autism Spectrum Disorder in children.
2. Vaccinations expose children to mercury through the use of thimerosal, which is a reason for the rise in Autism Spectrum Disorder.
3. Vaccinations can have serious side effects that cause more harm than some of the diseases that they are supposed to prevent.
4. Vaccines introduce toxic chemicals to the body that are not found in the natural immune defenses.
5. Even if people are vaccinated, there is still a risk of contracting the disease that the vaccination was intended to protect against.
6. Vaccinating a child before his/her immune system is fully developed can cause harm to that child.
7. An increase in hygiene and improved living conditions are the reasons why the prevalence of diseases has declined; it is not a result of an increase in vaccinations.
8. The use of aluminum in vaccinations is a risk for Alzheimer’s disease, dementia, and seizures.
9. Vaccinations do not lead to life-long immunity, whereas contracting a disease, such as chicken pox does result in life-long immunity due to the body’s natural defense mechanisms.
10. Due to political ties and economic incentives, pharmaceutical companies are not trustworthy sources to regulate the safety of vaccinations.
