# Supplementary material for: Beliefs about Vaccinations: Comparing a Sample from a Medical School to That from the General Population
Source: Int J Environ Res Public Health. 2018 Mar 28;15(4):620. doi: 10.3390/ijerph15040620 (PMC5923662; doi:10.3390/ijerph15040620)
Supplement: Supplementary file 1 [file ijerph-15-00620-s001.pdf]

Supplemental Material

Results for the General Statements

Agreement for General Statements

Each participants' overall agreement was calculated for the general statements. A value of 1 was given for each statement the participants claimed that they agreed with, and a value of 0 reflected no agreement with the statement. The average agreement was calculated by total agreement across the general statements divided by 20 statements. An ANOVA was used to determine if there were differences between the medical affiliates' (M = .69, SD = .08) and the general population's (M = .75, SD = .09) agreement among the general statements. A significant difference for the general statement agreement was found between the two groups $F(1, 233) = 20.78$, $p < .001$. Therefore, the general population agreed with more of the general statements than the medical affiliates. Additional data analysis regarding the agreement for the general statements will be presented after the results from the categorization of facts versus beliefs.

Categorization of Facts and Beliefs

Each participants' fact categorization was calculated for the 20 general statements. If participants selected that the statement was a fact, the statement received a score of a 1, whereas if the participants indicated that the statement was a belief, the statement received a score of a 0. The average of all the responses was determined to determine an average fact versus belief score for each statement. See Table 1 for the average fact versus belief scores for each general statement for the general population and the medical population. An ANOVA was conducted to determine if fact categorization for the general statements varied between the two populations. The medical affiliates and general population provided similar responses as to which statements were facts and which were beliefs (M = 0.48, SD = 0.04; M = 0.48, SD = 0.09, respectively). No significant difference was found between the two groups, $F(1, 233) = 0.34$, p = .854.

For the general statements, we observed that the general population agreed with the statements significantly more than the medical affiliates. To further examine the difference in fact categorization and agreement between the general population and the medical affiliates, additional statistics were computed to determine if agreement varied as a function of categorization. Overall, there was a significant effect on level of agreement between the statements categorized as beliefs and those categorized as facts, $F(1, 232)$ = 953.69, $p < .001$. Both populations were more likely to agree with the statements categorized as facts compared to beliefs. Independent t-tests were computed to evaluate whether agreement varied as a function of categorization. The general population were significantly more likely to agree with the statements and categorized the general statements as "beliefs" (M = 0.52, SD = .18) as compared to that of the medical affiliates (M = .42, SD = .16), $t(177) = -4.15$, $p < .001$. There was not a significant difference between the medical affiliates (M = .99, SD = .04) and the general population (M = .98, SD = .11) in relation to agreement among the statements that the participants indicated as "facts," $t(232) = 0.59$, p = .553.

Do People Agree as to what is a Fact and What is a Belief?

To determine if the medical and the general population had consensus, or agreed with each other as to which general statements were facts and beliefs, fact categorization scores were computed. Each statement by participant received a score of 1 if they categorized the statement as a fact, and a score of 0 if they categorized the statement as a belief, and the average score for each statement was computed.

Therefore, if all the participants indicated that the statements were "facts," the average fact categorization for the statement would be 1. An average score of 0 would infer perfect agreement that a statement is a belief. A score of 0.5 would indicate that 50% of participants categorized the statement as a belief and 50% categorized the statement as a fact; therefore, representing no consensus.

To determine if there was consensus for the statements, the average consensus values for each of the general statements were converted into scores that ranged from 0 to 0.4. These scores were obtained by assigning a value based on how far away a statement was from agreement (0 or 1). The number of statements that had average values ranging from 0-0.09 or from 0.9-1 were multiplied by 0. The number of statements that had average values between 0.1-0.19 and 0.8-0.89 were multiplied by 0.1. The number of statements that had average values ranging from 0.2-0.29 or 0.7-0.79 were multiplied by 0.2. The number of statements that had average values between 0.3-0.39 and 0.6-0.69 were multiplied by 0.3. Lastly, the number of statements that had average values ranging from 0.4-0.49 and 0.5-0.59 were multiplied by 0.4. If perfect consensus among all participants across all statements was achieved, the average consensus value would equal 0. To determine if the data represented true consensus, the consensus values were then compared to the expected value of 0.2 (i.e., the value associated with random selection). Consensus values that were significantly less than 0.2 were considered as representing consensus among the participants as to which statements were categorized as facts and which statements were characterized as beliefs (Rabinowitz et al., 2013).

Independent t-tests were calculated to see if the medical affiliates' average consensus and the general population's average consensus differed from 0.2. The medical affiliates displayed consensus in categorizing the general statements ($M = 0.01$, $SD = 0.03$), $t(57) = -27.61$, $p < .001$. The general population also displayed consensus in categorizing the general statements ($M = 0.02$, $SD = 0.04$), $t(177) = -22.58$, $p < .001$. See Figure 1 for the number of general statements plotted as a function of the average consensus values.

Accuracy of Predictions

Participants were asked to predict the percentage of people that would agree with each statement. The actual and predicted values of agreement among the general statements were calculated. Accuracy values were calculated by taking the difference between each participants' agreement estimates for the general population and the average of the agreement calculated from all the participants for each general statement. A correlation with Fisher correction was then calculated between the participants level of agreement and the accuracy of their prediction. This measure would show whether people were overestimating the level of agreement of their beliefs with that of others (the truly false consensus effect) or underestimated the level of agreement (the illusion of uniqueness).

Independent t-tests were computed for both the medical affiliates and the general population to observe if the accuracy values significantly differed from 0. Both the medical affiliates and the general population showed negative correlations ($M = -0.07$, $SD = 0.29$; $M = -0.17$, $SD = 0.34$, respectively) The accuracy values for the medical affiliates did not significantly differ from 0, $t(57) = -1.72$, $p = .091$; however, the general population's accuracy values were significantly inaccurate with their predictions of other's agreement, $t(176) = -6.75$, $p < .001$. This indicates that the general population underpredicted the amount of agreement with their perspectives. Another independent t-test was conducted to determine if there were significant differences in accuracy predictions between the two population groups. The general population significantly underestimated others' attitudes in relation to agreement compared to the medical affiliates' estimations, $t(233) = 2.11$, $p = 0.036$.

Table S1: Fact versus belief averages for each general statement by population type.

General Statements	Medical Affiliates *M*	General Population *M*
A hammer is used to pound nails.	0.90	0.94
Children are happy and carefree.	0.02	0.10
Dogs are animals.	0.98	0.95
A pen is for writing.	0.91	0.89
The telephone is the greatest invention of all time.	0.00	0.02
There are three colors in the American flag.	1.00	0.96
There are seven days in a week.	1.00	0.95
Christmas is a holiday primarily for children.	0.00	0.01
Sleeping with the windows is good for you.	0.02	0.01
Thermometers are used to record temperatures.	0.98	0.93
A driver's license is required by law for driving a car.	0.98	0.94
Books may be borrowed from the library.	0.88	0.96
Rich people are happy people.	0.02	0.02
Cats are friendly animals.	0.00	0.03
Rock music has a bad influence on young children.	0.00	0.01
It is okay to lie.	0.02	0.03
The longer you stay in school the smarter you will be.	0.02	0.06
The earth revolves around the sun.	0.98	0.93
Comic strips are funny.	0.00	0.06
The shape of a ball is round.	0.91	0.89

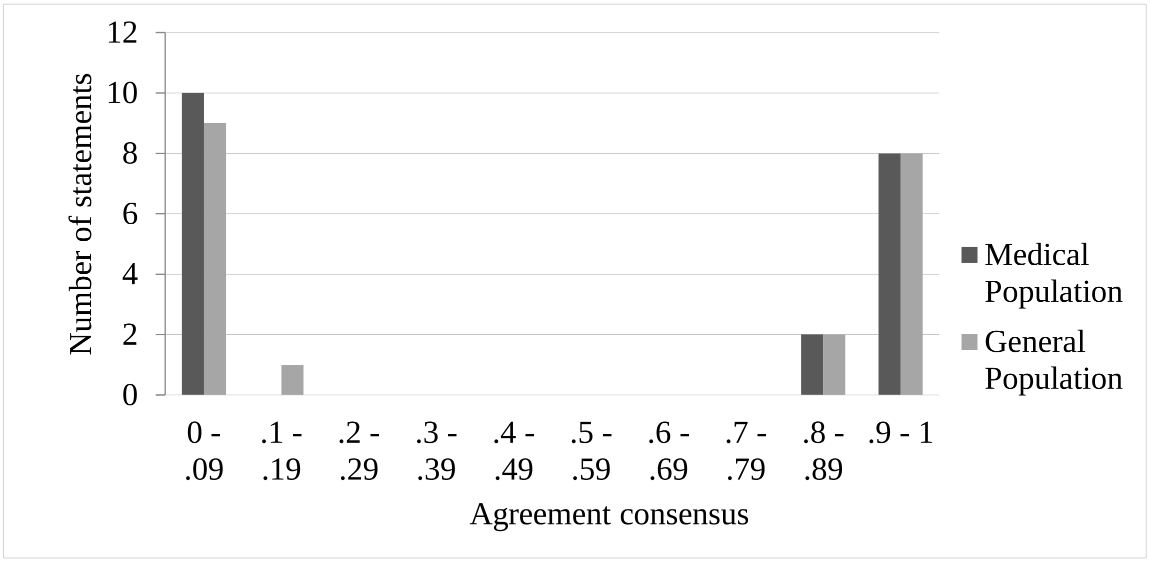

Figure S1: Number of statements as a function of average consensus for the general statements.
